# An EDucation and eXercise intervention for gluteal tendinopathy in an Irish setting: a protocol for a feasibility randomised clinical trial (LEAP-Ireland RCT)

**DOI:** 10.12688/hrbopenres.13796.1

**Published:** 2023-12-14

**Authors:** Sania Almousa, Bill Vicenzino, Rebecca Mellor, Alison Grimaldi, Kathleen Bennett, Frank Doyle, Geraldine M. McCarthy, Suzanne M. McDonough, Jennifer M. Ryan, Karen Lynch, Jan Sorensen, Helen P. French

**Affiliations:** 1 School of Physiotherapy, Royal College of Surgeons in Ireland, Dublin, Ireland; 2 School of Health and Rehabilitation Sciences, The University of Queensland, Saint Lucia, Queensland, Australia; 3 Division of Population Health Sciences, Royal College of Surgeons in Ireland, Dublin, Ireland; 4 Department of Health Psychology, Royal College of Surgeons in Ireland, Dublin, Ireland; 5 Department of Rheumatology, Mater Misericordiae University Hospital, Dublin, Ireland; 6 Patient Representative, Dublin, Ireland; 7 Health Outcomes Research Centre, Royal College of Surgeons in Ireland, Dublin, Ireland

**Keywords:** Gluteal tendinopathy, greater trochanteric pain syndrome, usual care, physiotherapy, education, exercise, clinical trial

## Abstract

**Background:**

Gluteal tendinopathy (GT) is a degenerative tendon condition characterised by pain over the greater trochanter of the hip. A randomised controlled trial (RCT) in Australia found that 14 sessions of EDucation on load management plus eXercise (EDX) delivered over 8 weeks resulted in greater improvements in global rating of change and pain outcomes at 8 and 52 weeks, compared with corticosteroid injection or ‘wait and see’. Typically, 5-6 physiotherapy sessions are provided in public and private physiotherapy settings in Ireland, therefore, the aim of this study is to examine the feasibility of conducting an RCT to investigate effectiveness of 6 sessions of the EDX programme compared to usual care.

**Methods:**

We will randomly allocate 64 participants with GT to physiotherapist-administered EDX or usual care. The EDX intervention (EDX-Ireland) will be delivered in 6 sessions over 8 weeks.

To determine feasibility of an RCT, we will assess recruitment and retention and outcome measure completion. The health status outcomes to be assessed at baseline, 8 weeks and 3 months include: Global Rating of Change, pain severity, the Victorian Institute of Sport Assessment-Gluteal Questionnaire (VISA-G), the Patient-Specific Functional Scale, the Pain Catastrophizing Scale, Patient Health Questionnaire (PHQ), Pain Self-Efficacy Questionnaire, the EQ-5D-5L, the Central Sensitisation Inventory and hip abductor muscle strength. We will explore acceptability of the EDX-Ireland intervention from the perspective of patients and treatment providers, and the perspective of referrers to the trial. A Study Within A Trial will be also applied to compare recording of exercise adherence using app-based technology to paper-based diary.

**Discussion:**

There is a need to establish effective treatments for GT that potentially can be implemented into existing health systems. The findings of this feasibility trial will inform development of a future definitive RCT.

**Registration:**

The trial is registered prospectively on ClinicalTrials.gov ( NCT05516563, 27/10/2022).

## Introduction

Gluteal Tendinopathy (GT) is a degenerative condition of the gluteus medius and minimus tendons characterised by lateral hip pain at or around the greater trochanter of the hip, with marked palpation tenderness ( Cook & Purdam, 2009). It is also known as Greater Trochanteric Pain Syndrome (GTPS). GTPS is an umbrella term used to describe pain over the greater trochanter of the lateral hip, primarily due to gluteus medius and/or minimus tendinopathy, with symptoms also potentially arising from the trochanteric bursa ( Kong *et al.*, 2007; Stephens *et al.*, 2019). However, the terminology of gluteal tendinopathy is used when gluteus medius and/or minimus tendon changes are detected on Magnetic Resonance Imaging (MRI) ( Dadour *et al.*, 2021). Trochanteric bursitis has been shown to be present in less than 20% of those with lateral hip pain ( Kong *et al.*, 2007).

GT is the most common lower limb tendinopathy and three times more prevalent in women than men, affecting up to 25% of women aged over 40 years ( Segal *et al.*, 2007). One-year period incidence has been reported as 1.8/1000 in general practice ( Riel *et al.,* 2019). GT can result in very high levels of hip dysfunction, with consequent negative impact on general health and well-being, quality of life and employment status, similar to levels associated with end stage hip OA ( Fearon *et al.*, 2014; Plinsinga *et al.*, 2018).

Along with structural changes in the gluteus medius and minimus tendons ( Connell *et al.*, 2003), individuals with GT have reduced hip abductor strength ( Allison *et al.*, 2016b) and higher external hip adduction moments during loading activities such as walking and stair-climbing ( Allison *et al.*, 2018; Allison *et al.*, 2016a).

Abnormal hip biomechanics may predispose the development of GT. Specifically, it has been hypothesised that gluteal tendinopathy occurs due to compressive impingement of the gluteal tendons and bursa onto the greater trochanter by the iliotibial band (ITB) as the hip moves into adduction. Compressive forces are increased due to potential hip abductor muscle weakness, resulting in lateral pelvic tilt or ipsilateral shift in single leg stance, and relative hip adduction ( Mellor *et al.*, 2016).

Although this evidence highlights the rationale for strengthening as a key intervention, traditionally, corticosteroid injections have been used to achieve pain relief. The lack of evidence for the long-term benefits of steroid injection highlights that interventions that result in long-term benefits are required ( Rompe *et al.*, 2009). Additionally, steroid injections can weaken tendons, making them more vulnerable to tears ( Kleinman & Gross, 1983; Park *et al.*, 2013).

There is general consensus, in line with management of other tendinopathies, that interventions should aim to reduce compressive and tensile loads through education on how to minimise or adapt compressive activities and positions such as single-leg stance (walking, running and stair climbing), adduction-related activities such as sitting with legs crossed, certain hip stretches and sleeping positions ( Cook & Purdam, 2009; Grimaldi & Fearon, 2015).

Although a range of conservative management approaches are generally recommended, best practice in the management of GTPS/gluteal tendinopathy remains unclear. A three-arm Randomised Controlled Trial (RCT) in Australia (LatErAl hiP pain (LEAP) trial) compared 14 sessions of EDucation on load management plus eXercise (EDX) over 8 weeks to a single corticosteroid injection (CSI), and a ‘wait and see’ comparison group on pain and global improvement in 204 individuals with GT. Results showed that at 8 weeks (completion of treatment), 77% of the EDX group were at least ‘moderately better’, compared with 58% in the injection group, and 29% in the wait-and-see group, with improvements maintained at one year ( Mellor *et al.*, 2018).

However, the number of physiotherapy treatments provided may not be feasible in some countries. For example, although exercise and education are the most common interventions for GT delivered by physiotherapists in Ireland, the number of physiotherapy sessions is usually limited to five or six ( French *et al.*, 2020). This treatment dosage is consistent across various musculoskeletal pathologies in the UK ( Bury & Littlewood, 2018). Thus, whilst the LEAP trial in Australia demonstrated positive effects for EDX, delivery of this intervention over 14 sessions may not be practical in other healthcare systems, and evaluation of a lower dose of face-to-face sessions with a healthcare practitioner, which could be implemented into clinical practice is warranted.

Although current best evidence indicates, based on the LEAP trial findings, that a combination of physiotherapist-delivered exercise and education is most effective for management of GT, there are currently no clinical guidelines to guide practice. Indeed, usual care of gluteal tendinopathy by medical practitioners, to whom patients may initially present, is currently unknown.

This feasibility RCT will contribute to the following gaps in the literature: 1) to examine the feasibility of an RCT to compare effectiveness of six sessions of physiotherapy-led Education on load management plus eXercise (EDX-Ireland) EDX to usual care and 2) to quantify usual care in GT management in Ireland.

Completion of a daily HEP is essential to optimise exercise benefits and is an integral part of the EDX-Ireland intervention. Traditionally, paper-based diaries are used to record adherence, but adherence can be over-reported compared with electronic diaries ( Stone *et al.,* 2003). An RCT demonstrated improved exercise adherence using an app in people with musculoskeletal conditions, compared to paper handouts ( Lambert *et al.*, 2017), with high satisfaction rates reported by those who used the app. Given that >90% of phone users in Ireland have smartphones ( Deloitte, 2023), the use of app-based technology may be potentially more feasible to monitor and enhance treatment adherence.

We therefore aim to evaluate the feasibility of conducting a definitive RCT, using a reduced dose (6 sessions) of a proven-effective physiotherapy treatment (14 sessions) consisting of EDucation plus eXercise for GT in an Irish context. A Study Within A Trial (SWAT) will compare smartphone application *versus* paper-based diary for exercise adherence measurement. A SWAT is a type of research study that has been embedded within a host larger trial to evaluate alternative ways of delivering or organising a particular trial process ( Treweek *et al.*, 2018).

### Objectives

Specific objectives are as follows:

1. To assess recruitment and retention rates to inform a future definitive RCT

2. To estimate the effect size for a likely primary study outcome in a future definitive RCT

3. To determine the success of different patient recruitment methods (community recruitment, primary care and secondary care)

4. To identify the constituents of 'usual care' for gluteal tendinopathy in an Irish context and to determine if inclusion of a usual care arm in a future definitive RCT is feasible and appropriate without the potential for co-intervention bias

5. To assess outcome measure completion

6. To assess the feasibility of collecting healthcare utilisation and cost data for cost-effectiveness analysis

7. To assess if adherence to the home exercise programme (HEP) for those allocated to the EDX-Ireland intervention differs when recorded *via* a smartphone application compared with paper diaries

8. To assess the acceptability of the EDX-Ireland intervention and usual care from the perspective of study participants and service providers (treating therapists and providers of usual care)

9. To assess participants’ and therapists’ acceptability of a smartphone-based application (app) for exercise provision and home exercise adherence recording in the EDX-Ireland arm

10. To compare EDX with usual care on clinical outcomes including pain, function, hip abductor muscle strength, psychological measures, and quality of life.

## Methods

### Design and study setting

**Figure 1.**
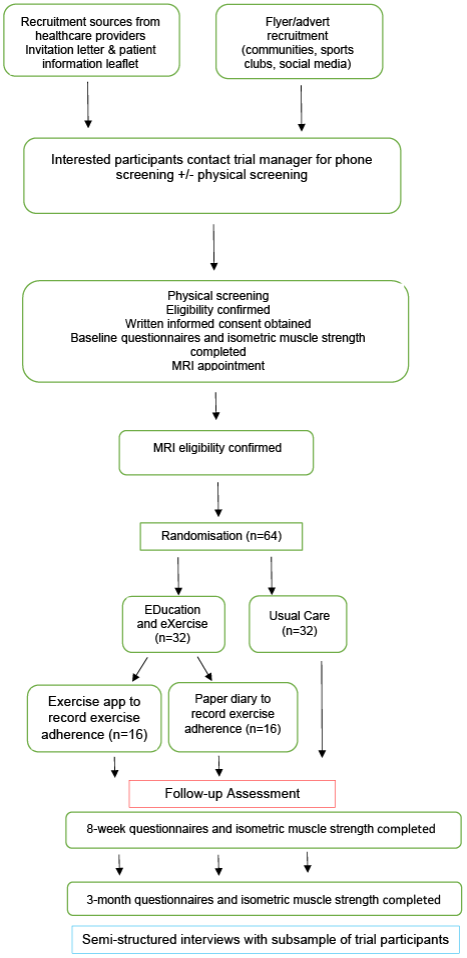
Overview of LEAP-Ireland procedures. LEAP-Ireland, LatErAl hiP pain project; MRI, Magnetic Resonance Imaging.

This feasibility, assessor-blinded RCT will take place in Dublin, Ireland. Telephone and physical screening will take place at RCSI University of Medicine and Health Sciences, while the EDX-Ireland intervention will be delivered in four physiotherapy clinics located in various northside and southside Dublin locations. The trial protocol follows the Standard Protocol Items: Recommendations for Interventional trials (SPIRIT) guidelines adapted for the reporting of protocol manuscripts of pilot and feasibility trials ( Chan *et al.*, 2013; French & Almousa, 2023b). An overview of the LEAP-Ireland procedure is outlined in Figure 1. The model consent form can be found as *Extended data* ( French & Almousa, 2023a). The trial is registered prospectively on ClinicalTrials.gov ( NCT05516563, 27/10/2022).

### Ethics approval

Ethics approval for this study has been granted by Research Ethics Committee of the Royal College of Surgeons in Ireland (RCSI) (REC202205010) on 19/08/2022, Irish College of General Practitioners (ICGP) (ICGP_REC_22_012) on 28/06/2022, Mater Misericordiae University Hospital (1/378/2303) on 25/10/2022, Beaumont Hospital (22/29) on 07/03/2023 and St Vincent's University Hospital (RC22-023) on 13/10/2022.

### Participants

As the EDX programme in this trial has been designed specifically to address gluteal tendinopathy, other differential diagnoses for lateral hip pain such as hip osteoarthritis are excluded ( Woodley *et al.*, 2008) thus, various eligibility criteria will be applied.

Table 1 outlines the inclusion and exclusion criteria, which are based on the LEAP trial ( Mellor *et al.*, 2018). 

**Table 1.** Inclusion and exclusion criteria.

Inclusion Criteria	Exclusion Criteria
• Aged 35–70 years • Lateral hip pain for at least 3 months, of ≥ 4/10 on an 11- point NPRS on most days of the last 3 months • Tenderness on palpation of the greater trochanter • Reproduction of pain on at least one of following diagnostic clinical tests: (FADER test, FADER with static muscle test (internal rotation) at end of range (FADER- R), FABER test, passive hip Adduction in side-lying (ADD) test, adduction with resisted isometric abduction (ADD-R), and single leg stand (SLS) for 30 seconds • Demonstrated tendon pathology on MRI	• Previous cortisone injection in the region of the lateral hip in the last 12 months • Physiotherapy (including regular Physiotherapy-led Pilates) for lateral hip pain in the last 3 months • Lumbar spine or lower limb surgery in the last 6 months • Any known advanced hip joint pathology where groin pain is the primary complaint and/or where groin pain is experienced at an average intensity of ≥2 on most days of the week, or Kellgren-Lawrence score of >2 (mild) on X-Ray • Clinical criteria for the diagnosis of hip osteoarthritis (Altman *et al.*, 1991) ○ Self-reported hip pain with either hip internal rotation <15° and hip flexion ≤115°, or ≥15° hip internal rotation and pain on hip internal rotation ○ Morning stiffness ≤ 60 minutes ○ Age ≥ 50 years ○ Hip joint flexion <90°, bilaterally • Lumbar radiculopathy or pain in another body location greater than the hip pain • Known advanced knee pathology or restricted knee range of motion (must have minimum 90° flexion and full extension, bilaterally) • Any systemic diseases affecting the muscular or nervous system, or uncontrolled diabetes • Fibromyalgia • Use of walking aid • Malignant tumour (current or in the past 6 months) • Systemic inflammatory disease • Any factors that preclude the participant from having an MRI (e.g., pacemaker, metal implants, pregnancy, or trying to become pregnant, claustrophobia) • If the participant is involved in any injury claim • If the participant is unable to commit to an 8-week programme of up to 6 sessions of exercise • If the participant is unable to write, read or comprehend English • Unable or unwilling to use technology for exercise prescription and adherence

FABER, Flexion, Abduction, External Rotation; FADER, Flexion, Adduction, External Rotation; MRI, Magnetic Resonance Imaging; NPRS, Numerical Pain Rating Scale.

### Recruitment

Participants will be recruited from various sources, including rheumatology, orthopaedic and musculoskeletal triage clinics in three acute hospitals in Dublin, General Practitioners (GPs), social media and community recruitment throughout Dublin *via* advertisements in sports and leisure clubs and community organisations in Dublin.

### Procedure

Initial screening for eligibility will be done *via* telephone by the Trial Manager (SA). If the phone interview indicates possible eligibility, potential participants will undergo a physical examination against specific selection criteria, undertaken by the Trial Manager. A battery of clinical tests will be used at the physical screening stage to clinically diagnose GT ( Grimaldi *et al.*, 2017). To be eligible, participants must experience pain on direct palpation of the gluteal tendon insertion on the greater trochanter, with reproduction of lateral hip pain on at least one of the following clinical tests: Hip Flexion, Adduction, External Rotation (FADER), resisted internal rotation in the FADER position (FADER-R), Flexion, Abduction, External Rotation (FABER), passive hip adduction in side lying (ADD), resisted isometric abduction in the ADD test position ADD-R), and a Single Leg Stance on the affected leg for 30 seconds ( Mellor *et al.*, 2016). Participants deemed eligible based on the clinical tests will then be referred for MRI to confirm the tendinopathic changes on MRI. Criteria for tendon pathology on MRI are described in Table 2.

**Table 2.** MRI classification of pathology for definition of gluteal tendinopathy ( Mellor *et al.*, 2016).

T2 Hyperintensity around Greater Trochanter (representing oedema/fluid	
**Size**	(1) Tiny (thin slit of fluid) (2) Small (localized, mild distension) (3) Medium (localized, moderate distension) (4) Large (localized, marked distension)
**Shape**	(1) Feathery (2) Crescentic (3) Round (distended bursa)
**Location**	(1) Subtendinous (2) Intratendinous * (3) Subfascia lata (4) Superficial to fascia lata
**Partial thickness tear**	Tendon irregular, thinned or focally Discontinuous
**Full thickness tear**	Discontinuity and/or retraction of the torn tendon from greater trochanter

*Intratendinous high T2 signal considered as tendinopathy with a thickened tendon without any irregularity, tendon thinning, or focal tendon discontinuity. MRI, Magnetic Resonance Imaging.

Following phone, physical screening and imaging will be conducted to confirm eligibility. On attending the physical screening, the Trial manager will answer any queries and collect the signed and completed consent forms. Once participants complete the baseline assessment, they will be randomly allocated into one of two groups: (1) education and exercise programme (EDX-Ireland), or (2) usual care. Participants will complete the patient-reported outcomes (PROs) and abductor muscle strength assessment at baseline, 8 weeks, and 3 months.

Participants will be asked to refrain from other treatments during the trial period, but analgesia and anti-inflammatory drugs will be permitted. All medication use and co-interventions will be recorded *via* the healthcare utilisation questionnaire.

### Blinding

The trial manager (SA) assessing outcome measures at baseline, 8 weeks and 3 months follow-up will be blinded to group allocation and will not be involved in randomisation or intervention delivery.

### Randomisation and Concealed allocation

The randomisation schedule will be generated using a computer-generated random sequence in advance of trial commencement by the trial statistician (KB), who will not be involved in recruitment or outcome assessment. Randomisation will be stratified by recruitment method (GP, secondary care or community/social media). Participants will be randomly allocated (1:1 ratio) using random blocks of size 2, 4, 6, or 8 within each stratum. Within the intervention arm (n=32) a further 1:1 blocked randomisation (block size 2, 4 or 6) will be developed for random allocation of the EDX-Ireland group, within the SWAT, to either the smartphone application (n=16) or paper-based diaries (n=16). Randomisation and allocation will be done by the PI (HPF), who will not be involved in screening or baseline assessment of trial participants, thus maintaining concealed allocation.

### Interventions

The EDX intervention is described based on the Template for Intervention Description and Replication (TIDieR) ( Hoffmann *et al.*, 2014).

The EDX-Ireland intervention will be delivered in four physiotherapy clinics located in northside and southside Dublin locations. Physiotherapists will have attended a training session outlining study objectives and requirements, demonstrating the detailed education material, the theoretical and practical part of the exercise protocol and progressions. Additionally, a detailed study manual will be provided for reference ( Mellor *et al.*, 2016).

The EDX-Ireland intervention will involve six face-to-face sessions with a physiotherapist, delivered over eight weeks (60 mins for the initial session and 30 mins thereafter). The sessions will be scheduled as weekly appointments for the first four weeks and the final two sessions will be delivered at fortnightly intervals. Participants will receive education on tendon care and gradual progression of tendon loading. The treating physiotherapist will also provide graduated exercise with 4-6 exercises performed six days a week, with alternate low and high load days, and one rest day. The exercise programme includes three key streams:

1. Isometric abduction: low load isometric abduction training in supine and standing, to improve muscle coordination and control, and to potentially assist with pain relief.

2. Functional loading: bridging (double-leg, offset and single-leg exercises) and squatting (double-leg, offset, single-leg stance and squat, step-ups) progressions with an emphasis on gluteal muscle recruitment and femoropelvic control.

3. Abductor loading: targeted frontal plane abductor muscle and tendon loading to improve load tolerance by applying progressively higher loads across the abductor muscles, as tolerated. This includes sidestepping tasks and standing band-resisted hip abduction.

Week 1 will focus on familiarisation with the basic program and load management strategies. Week 2 will involve early loading and optimisation of movement patterns in the base exercises. Weeks 3-8 focus on graduated loading, where participants will perform exercises, six days a week, with three of those days including exercises performed at a 'hard' to 'very hard' level. These hard days will alternate with three ‘light’ days’ and one rest day. The physiotherapist will monitor exercise difficulty level, exercise technique and pain response to exercise. Level of exercise difficulty will be monitored with the modified Borg Scale of Perceived Exertion (0-10 scale), where isometric abduction will be performed at a 'light' level (Borg 1-2), functional loading at a 'somewhat hard' to 'hard' level (4-6), and targeted abductor loading from 'somewhat hard' towards 'hard' to 'very hard' level (7-9), depending on response to loading.

### Study Within A trial (SWAT)

To test objective 7, the 32 participants randomly allocated to the EDX-Ireland intervention will also be randomly allocated to either a ‘PhysiApp’ or ‘Diary’ group for recording of home exercises, using a second computer-generated random sequence, prepared by the trial statistician. Participants will be informed at the time of group allocation by the randomiser (HPF). Treating therapists will be provided with access to Physitrack (Physitrack Ltd, London, UK), an online exercise management system used by physiotherapists for exercise prescription for the duration of the recruitment and intervention period. Both groups (‘PhysiApp’ and ‘Diary’) will use the patient-facing companion app, PhysiApp (Physitrack Ltd, London, UK), for home exercises, but only the ‘PhysiApp’ group will record exercise adherence on the app. Those assigned to the ‘Diary’ group will be provided with a paper-based diary to record exercise completion and the exercise recording function in PhysiApp will be deactivated. Physiotherapists will check adherence at each appointment and assist with any barriers to exercise completion.

### Usual care

Usual care was chosen for this study as the comparator to determine if it is an appropriate comparator, and to identify the exact constituents of usual care, in advance of conducting a full-scale RCT. Usual care for this trial is defined as the treatment that a participant has followed so far or has been prescribed by their referrer into the trial ( *i.e.*, GP, orthopaedic consultant, rheumatologist or musculoskeletal triage physiotherapist). For those not referred from a healthcare practitioner ( *e.g.*, community recruitment/social media), usual care may not be relevant, but any healthcare utilisation will be recorded on a pre-standardised form. Participants randomised to the Usual Care group will receive an information leaflet on their pathology and general advice on symptom management.

### Intervention acceptability

To further explore trial feasibility and acceptability (objectives 8 and 9), semi-structured interviews will be conducted with approximately 18 participants (recruited from both groups) to explore their experiences of the trial, intervention content, face-to-face clinic interaction, HEP completion, and acceptability of the smartphone app or paper diaries for HEP recording. The topic guides will be developed using the Theoretical Framework of Acceptability ( Sekhon *et al.*, 2017). Semi-structured interviews will also be conducted with treating therapists to ascertain their perspectives of the acceptability of the EDX-Ireland intervention. Interviews will also be undertaken with healthcare professionals (doctors or musculoskeletal triage physiotherapists) who referred participants into the trial to explore their knowledge of management options for gluteal tendinopathy, their rationale for choice of usual care, as well as any barriers or facilitators to providing optimal care for gluteal tendinopathy. Interviews will be audio recorded and transcribed verbatim for thematic analysis.

### Outcomes measures

***Primary outcome measures.*** Primary outcomes focus on feasibility issues including success of recruitment and retention to the 3-month time point. Details of feasibility outcomes and methods used to collect these outcomes are outlined in Table 3.

**Table 3.** Feasibility outcomes.

Objective	Data collection method
To assess recruitment rates and determine the success of different patient recruitment methods community recruitment, primary care and secondary care) To assess short (8-week) and medium-term (3-month) retention rates	• Number of individuals who contact the research team about the trial (community recruitment) • Recruitment source of each person who undergoes screening process • Number who do/do not consent to telephone screening • Number who are eligible/ineligible to proceed to physical screening • Number who are eligible/ineligible to proceed to MRI • Number who are eligible/ineligible based on MRI findings • Number who are eligible based on MRI findings but decline to participate • Number who consent to participate in the study • Number randomised to participate in the study • Number who attend for 8-week and 3-monthfollow-up • Number who cannot be contacted • Number who do not attend • Number who cannot attend/cancel follow-up (reasons)
To identify the constituents of ‘usual care’ and to determine if inclusion of a usual care arm is feasible and appropriate without the potential for co- intervention bias	Referral sources will be asked to complete a checklist of usual care options (including medications, referral for physiotherapy/other healthcare services). This will be cross-referenced against the participant-completed health and cost questionnaire. Participants will also complete details on current and past treatment for gluteal tendinopathy at baseline assessment.
To estimate the effect size for a likely primary study outcome in a future definitive trial	Based on clinical outcomes collected at 8 weeks and 3 months
To determine outcome measure completion	Completion rate of clinical outcomes collected at baseline, 8 weeks and 3 months
To assess the feasibility of collecting healthcare utilisation and cost data for future cost-effectiveness analysis in a definitive trial	Participants will complete questionnaires on use of pain medications, visits to GP and other medical specialties, investigations, attendance at physiotherapy or other healthcare practitioners or, hospital attendance for their hip pain until the 3-month follow-up
To assess if adherence to the home exercise programme of those allocated to EDX-Ireland differs when recorded on a smartphone application compared with paper diaries	Adherence to the home exercise programme will be recorded by the study participant using the smartphone app or paper-based diary
To assess participants’ and therapists’ acceptability of a smartphone-based application (app) for recording home exercise Adherence in the EDX arm To assess the acceptability of the EDX intervention and usual care from the perspective of study participants and service providers (treating therapists and providers of usual care)	Semi-structured interviews of study participants to explore acceptability of the paper diaries and smartphone app for exercise provision and adherence recording

MRI, Magnetic Resonance Imaging; EDX, EDucation and eXercise.

***Secondary clinical outcomes.*** The following secondary outcomes, which are aligned to core domains recommended for use in tendinopathy research ( Vicenzino *et al.*, 2019) will be assessed at baseline, 8 weeks and 3 months.

*The Global Rating of Change (GROC)* is a 15-point rating scale, completed by the patient based on their perceived overall change in their hip condition and ranges from –7(a very great deal worse) to +7 (a very great deal better) ( Kamper *et al.*, 2009) and allows patients to consider factors that they consider important for their clinical situation.

Pain severity will be measured using a 11-point *Numerical Pain Rating scale (NPRS)* under two conditions of pain; on activity/loading and pain in the last week. It is reliable, valid and easy to administer. A minimal important difference (MID) of 1.5 points or 15% has been ascertained for musculoskeletal pain ( Abbott & Schmitt, 2014).

*Victorian Institute of Sport Assessment-Gluteal (VISA-G)* is a self-reported questionnaire, which measures disability associated with gluteal tendinopathy. It contains eight items, rated on an 11-point scale, related to pain and function and is reliable and valid ( Fearon *et al.*, 2015).

*The Patient-Specific Functional Scale (PSFS)* is a self-reported, patient-specific measure, designed to assess functional change. It is reliable and valid across various musculoskeletal disorders ( Abbott & Schmitt, 2014).

Psychological distress, which is associated with gluteal tendinopathy ( Plinsinga *et al.*, 2018; Plinsinga *et al.,* 2020) will be evaluated using two measures. The *Pain Catastrophising Scale (PCS)* is a valid and reliable 13-item self-report measure, which asks about thoughts and feelings associated with pain ( Sullivan *et al.*, 1995). The *Patient Health Questionnaire (PHQ)* is a valid and reliable self-reported questionnaire for measurement of depressive symptoms. It contains nine questions with a score range of 0-27. A score of ≥5 indicates mild depressive symptoms, ≥10 moderate, ≥15 moderately severe and ≥20 severe ( Kroenke *et al.*, 2001).

The *Pain Self-Efficacy Questionnaire (PSEQ)* assesses people’s confidence in performing activities when they are in pain ( Nicholas, 2007). It contains 10 items answered on a 7-point Likert scale.

*The EQ-5D-5L* is a generic measure of health-related quality of life (HR-QOL). Each health state is ranked and transformed into a single index score, called the utility score. This utility score will be used to express the Quality-Adjusted Life Years (QALY), which is commonly used in analyses of cost-effectiveness ( Rabin & de Charro, 2001).

*The Central Sensitisation Inventory (CSI)* is a 25-question self-reported screening instrument designed to identify presence of centrally-mediated pain sensitisation. A cut-off score of ‘40’ of ‘100’ yielded good (81%) sensitivity ( Neblett *et al.*, 2015).

*Isometric Hip Abductor Muscle Strength* will be tested in supine using a hand-held dynamometer (MicroFET ^®^2 Digital Handheld Dynamometer (Hoggan Scientific, Utah, USA). The leg will be positioned to minimise compression of the gluteal tendons against the greater trochanter. A non-elastic fixation belt will be placed around the patient’s pelvis and secured to the bed to minimise any trunk/pelvic movement during testing ( Allison *et al.*, 2016b). Pain severity using a 0-10 NPRS will be assessed immediately before and during strength testing to ascertain the impact pain may have on maximal effort.

Healthcare use and costs will be measured using a modified version of the *Osteoarthritis Cost and Consequences Questionnaire (OCC-Q)* ( Pinto *et al.,* 2011; Wilson *et al.*, 2023).

All assessments will take place at RCSI. Participants will complete PROs using Research Data Capture (REDcap) online electronic data capture tool ( Harris *et al.*, 2019), hosted at RCSI, for the baseline, 8-week and 3-month follow-up.

### Adverse events

Adverse events related to the EDX intervention are considered low risk in this trial. Treating physiotherapists will record and report adverse effects to the Principal Investigator. Serious adverse events will be reported to the trial sponsor and may result in trial withdrawal.

### Participant confidentiality and data management

Participants will be identified by a study-specific unique identifier number (UIN) during the study. Processing of any personal data will comply with national and EU General Data Protection Regulations (GDPR), and compliance with GDPR will be maintained throughout the trial.

### Statistical analysis

Descriptive analysis will include proportions and 95% confidence intervals (CI), means and standard deviations (SD) or medians and inter-quartile ranges (IQR) where appropriate. Feasibility outcomes of recruitment and retention rates will be descriptively reported overall across both arms of the trial (percentages and 95% CIs) and by method of recruitment. Outcome measure completion will be described both by participant and by outcome measure using percentages. Recruitment rates will be calculated as the number of participants who consent to participate divided by the number of eligible participants approached. Trial retention rates will be calculated from the number of participants who complete all outcome measures divided by the number who record baseline outcome measures. Adherence rate of exercises within the SWAT for the EDX group, and types of usual care for the control group will be reported as percentages.

Results of secondary clinical outcomes including NPRS, hip abduction strength and measures of psychological distress will be reported using means and SD or medians and IQR, where appropriate for baseline, 8- and 3- month follow-up. Statistical analysis will be performed using Stata v17 (StataCorp, College Station, Texas, USA).

Sensitivity analysis will be conducted as a secondary analysis to impute any missing data using multiple imputation. The resource use data captured by the OCC-Q will be valued using unit costs derived from local and national sources and will be analysed in a manner consistent with the clinical outcome data.

Qualitative data from interviews will be transcribed verbatim. A two-stage analysis will be undertaken, initially through inductive line-by-line coding of the extracted results to identify emergent themes, followed by deductive mapping of pre-identified codes to the Theoretical Framework of Acceptability ( Sekhon *et al.*, 2017).

Trustworthiness will be attained through procedures such as member checking, maintaining a reflexive journal of the research procedures and a clear audit trail of the process ( Braun & Clarke, 2014).

### Sample size

We will aim to recruit 64 participants. A sample of 60 participants (n=30 per arm of the trial) would provide an estimate of the recruitment rate of ≥50% (95% confidence interval (95% CI) 37.3% to 62.7%), and an estimated retention rate of ≥80% (95% CI of 69.8% to 90.2%). To ensure balance within each stratum, sample size will be increased to 64 with 32 allocated to each arm. This sample size will also allow for the potential effect of the primary outcome (GROC) between the two groups to be determined for powering a larger definitive trial.

### Study status

Study is currently ongoing. The expected end date for the study is July 2024.

### Patient and Public Involvement

This study embeds Patient and Public Involvement (PPI) in its overall approach and processes. To date, PPI actions have included contribution to outcome measures, study logo, recruitment strategies and participant-facing information regarding study recruitment materials.

### Trial governance and data management

This study will be conducted in accordance with international standards of Good Clinical Practice in trials. A Trial Management Committee (TMC) and a Trial Steering Committee (TSC) will be established to ensure adequate trial management, governance and safety monitoring for the trial. The Trial Management Group will be responsible for data management and stewardship, under the leadership of the principal investigator.

All data will be handled in accordance with RCSI research policies and ethical principles. The FAIR principles will be adhered to, to ensure Findability, Accessibility, Interoperability and Reusability. Research data will be stored on a unique SharePoint site at RCSI, managed, processed, and stored in a secure environment, and regularly and securely backed up by the RCSI IT department. Any data will be encrypted at source and transferred using secure methods that comply with RCSI data management policies and data protection policies for storage on the RCSI SharePoint site. Access to research files will be restricted to the research team. Data will be stored for a minimum of five years in line with HRB and RCSI guidelines on good research practice and general audit requirements.

### Dissemination of findings

We will disseminate study findings to scientific, lay and clinical audiences at local, national and international level through conferences, peer-reviewed journals and social media.

### Trial Sponsor

RCSI University of Medicine and Health Sciences ( sponsorship@rcsi.ie).

## Discussion

Gluteal tendinopathy is the most common lower limb tendinopathy, which can negatively impact everyday function and quality of life. We aim to evaluate the feasibility of a future definitive RCT to test the delivery of a lower dose (6 sessions) of a 14-session education and exercise programme, which demonstrated short and long-term improvement compared with corticosteroid injection and ‘wait-and-see’ ( Mellor *et al.*, 2018). Current evidence suggests that GT management needs targeted interventions, which improve the ability of the gluteus medius and minimus tendons to tolerate load required for everyday activities, and to strengthen the associated muscles, as well as minimising compressive load.

Exercise and load management are recommended as the cornerstone of an effective non-invasive management of tendinopathy ( Cook *et al.*, 2016). The exercise and load management programme to be used in this study is focused specifically on hip abductor muscle function, commencing at low load and gradually progressing over the course of the 8-week period, as well as avoiding compressive loads on the gluteal tendons, such as adopted/prescribed hip muscle stretching techniques ( Mellor *et al.*, 2016).

Additionally, this study will quantify current usual care for this condition. Whilst numerous systematic reviews of interventions for GT/GTPS have been published, no clinical guidelines have been developed to provide guidance on what usual care for this condition should include ( Barratt *et al.*, 2017; Gazendam *et al.*, 2022; Koulischer *et al.*, 2017). Usual care is a common comparator in intervention trials, and whilst it is a term used to describe current treatments used by clinicians, it is commonly unclear how it is defined and may be inconsistent, highly variable and non-evidence based ( Angriman *et al.*, 2019; Pascoe *et al.*, 2022). Findings from this feasibility trial should elucidate what typical care entails across primary and secondary care settings and may be used to inform decisions for comparators for a future definitive RCT.

## Data Availability

No data are associated with this article. Zenodo: Model Consent Form. https://zenodo.org/doi/10.5281/zenodo.10071858 ( French & Almousa, 2023a). Zenodo: SPIRIT checklist for ‘An EDucation and eXercise intervention for gluteal tendinopathy in an Irish setting: a protocol for a feasibility randomised clinical trial (LEAP-Ireland RCT)’. https://zenodo.org/doi/10.5281/zenodo.10093259 ( French & Almousa, 2023b). Data are available under the terms of the Creative Commons Attribution 4.0 International license (CC-BY 4.0).
