# Supplementary material for: An EDucation and eXercise intervention for gluteal tendinopathy in an Irish setting: a protocol for a feasibility randomised clinical trial (LEAP-Ireland RCT)
Source: HRB Open Res. 2024 Jun 28;6:76. Originally published 2023 Dec 14. [Version 2] doi: 10.12688/hrbopenres.13796.2 (PMC11263908; doi:10.12688/hrbopenres.13796.2)
Supplement: Peer review history

# Reviewer report: Reviewer response for version 2

**DOI:** 10.21956/hrbopenres.15234.r41035

**Published:** 2024-08-26

**Authors:** Angela Fearon

**Affiliations:** 1 University of Canberra, Canberra, Australia

I approve these changes and wait in eager anticipation to see the results of this trail. Well done team.

Is the study design appropriate for the research question?

Yes

Is the rationale for, and objectives of, the study clearly described?

Partly

Are sufficient details of the methods provided to allow replication by others?

No

Are the datasets clearly presented in a useable and accessible format?

Not applicable

Reviewer Expertise:

Musculoskeletal research, specifically around gluteal tendinopathy.

I confirm that I have read this submission and believe that I have an appropriate level of expertise to confirm that it is of an acceptable scientific standard.

# Reviewer report: Reviewer response for version 2

**DOI:** 10.21956/hrbopenres.15234.r39158

**Published:** 2024-07-22

**Authors:** Lyndsay A. Alexander

**Affiliations:** 1 School of Health Sciences, Robert Gordon University, Aberdeen, UK

Thank you for the invitation to review this protocol for a feasibility RCT adapting the LEAP trial to an Irish setting.

 The study rationale, objectives and study design are appropriate, and clearly described.

 The authors have addressed previous reviewer comments so i have only one main thought in relation to this paper:

 Within the methods there is a lack of clarity regarding inclusion of participants that may or may not own/have access to a smart phone. This is not stated in the inc/exc criteria nor does the text clarify if provision of a smart phone will be provided for the duration of the study. This may potentially impact inclusion for some and overall recruitment perhaps.

Is the study design appropriate for the research question?

Yes

Is the rationale for, and objectives of, the study clearly described?

Yes

Are sufficient details of the methods provided to allow replication by others?

Partly

Are the datasets clearly presented in a useable and accessible format?

Not applicable

Reviewer Expertise:

tendinopathy

I confirm that I have read this submission and believe that I have an appropriate level of expertise to confirm that it is of an acceptable scientific standard.

# Reviewer report: Reviewer response for version 2

**DOI:** 10.21956/hrbopenres.15234.r41034

**Published:** 2024-07-10

**Authors:** Chris Clifford, Katie Monnington

**Affiliations:** 1 Glasgow Caledonian University, Glasgow, UK; 2 Royal National Orthopaedic Hospital NHS Trust, London, UK

No further comments

Is the study design appropriate for the research question?

Yes

Is the rationale for, and objectives of, the study clearly described?

Yes

Are sufficient details of the methods provided to allow replication by others?

Yes

Are the datasets clearly presented in a useable and accessible format?

Not applicable

Reviewer Expertise:

Tendinopathy

We confirm that we have read this submission and believe that we have an appropriate level of expertise to confirm that it is of an acceptable scientific standard.

# Reviewer report: Reviewer response for version 1

**DOI:** 10.21956/hrbopenres.15095.r38734

**Published:** 2024-04-11

**Authors:** Angela Fearon

**Affiliations:** 1 University of Canberra, Canberra, Australia

The authors plan to test the feasibility of conduction an RCT into the effectiveness of an amended LEAP protocol, 6 sessions over 8 weeks, compared to the original of 14 session.  This is a very worthwhile project. 

 I do have some relatively minor concerns about the written protocol. 

Paragraph 1.

 1. Cook and Purdam (2009).  Defining GT is difficult, and I don't under-estimate the complexity of being clear with this definition. However, Cook and Purdams' 2009 paper does not provide a definition of GT. It provides a model to understand the clinical presentation and the underlying pathology - so as to guide treatment. Cook and Purdam make no reference to GT or even palpation. I suggest you consider...[1],[2],[3],[4].

 2.Kong et al 2006 (cited x2), is a descriptive paper.  There is no statement about how many participants were included, how they were chosen, what their clinical features were, or any information about the people who did the reports. It is not an incidence/prevalence paper. The papers above and [5], plus others you should look for, should help. 

 3. Stephens 2019 is a study on how physios treat people with GTPS. This also is not an appropriate reference for this statement. 

 4. I think you can find a better paper than Dodour et al 2021 for this statement. 

 Please check all your references from here forward. You should plan to use high quality primary references were possible to support your statements. 

Paragraph 2

 "GT is *(arguably)* the most common lower limb tendinopathy..." reference please (Abler 2016)

 ...in women at risk of knee oa  (the 25% statistic is a common error if based on Segal alone - 

 you need another citation for this statement - there are plenty out there. 

 Mellor et al 2016 - is the protocol for Mellor et al 2018 - only cite the latter. Further - this is the wrong paper (the Allison papers at the ones to look at). 

Paragraph 3

 "Although this evidence highlights the rationale for strengthening as a key intervention,"  Except that Mellor et al 2018 didn't demonstrate that a group difference in strength was associated with reduced pain. Consider rephrasing this in light of what Mellor et al 2018 actually report about the strengthening. 

 I'm not sure why you solely reference Rompe here - as the Mellor paper (and the Ganderton paper, and the Nissen paper) are more robust RCTs to reference. 

 Park 2013 is about muscles and fat. Kleinman and Gross is about ruptures - there are other papers that you will find that would be better references for this. 

Paragraph staring "However, the number..."

 Make it clear you are referring back to the LEAP trial - and reference it. 

 14 treatments is not likely to be practical in the Australian setting as well. Please don't assume that Australians will have 14 treatments. The French paper demonstrates this is. Thus, please revise. 

Paragraph starting "Although...:"

 It would be generous to include other studies that have had a positive result from exercise and education in this statement.

Completion of a (don't forget to check you have the best references here)

 define HEP

 Please provide a citation for "...is essential to optimise..."

We therefore aim to evaluate..

 You can't have a feasibility study and conduct a "definitive RCT" - please rephrase. 

Objectives

 2 - What is the likely primary outcome? 

Figure 1

 I am concerned that you have "+/- physical screening" in the box with the phone screening. I know from personal experience you can have a group of people with GT (and without some co-morbidities) without physical screening. 

Participants

 Note- the pain reproduction should be lateral, some people will get medial pain with these tests, indicating intra-articular joint pathology. Please clarify. 

 Consider the following reference for your diagnosis of hip OA[6]

 Table 2 - consider using the primary research here, rather than referencing a reference. Mellor et al didn't do the research to support this diagnosis - reference who did. 

Following phone... and Blinding

 This paragraph is awkward - please rephrase it. 

Interventions

 "The EDX-Ireland intervention will be delivered in four physiotherapy clinics located in northside and southside Dublin locations."

 Is this eight in total? (I don't think so, but it reads that way)

Week 1 will focus on...

 What will the physios do if the pain is exacerbated? Is there an escape clause? 

 Are there any guidelines around the advice the physios provide regarding barriers to completion? 

 Leaflet - is that included in the protocol? (there is no reference here and I can't see it in an appendix)

Acceptability

 Will you recruit equal numbers from both patient groups? 

 How many health care professionals are you planning on interviewing?  I ask b/c a) you should have a plan, and b) if you interview all the referring health care providers, you may end up with an unmanageable number. Thus, you need to plan how many, and how you'll decide who to interview. 

Health costs - will you be modifying this? and if so, what will the modifications be? Please provide some guidance around this, or include the modified version in an appendix. 

Adverse events

 Do you need to report these to your local ethics committee? 

Confidentiality

 Please provide a citation for the GDPR

Sample size

 I'm sorry, but I am unclear how you estimate the recruitment rate, without an estimate of how many people you think will be referred. 

Discussion

 Reference first paragraph appropriately

Additionally...note this is in Ireland. 

 Please provide in appendices information that would allow this trial to be repeated. 

 e.g. leaflets, noting if participants will get DVDs (as in the LEAP trial).

Is the study design appropriate for the research question?

Yes

Is the rationale for, and objectives of, the study clearly described?

Partly

Are sufficient details of the methods provided to allow replication by others?

No

Are the datasets clearly presented in a useable and accessible format?

Not applicable

Reviewer Expertise:

Musculoskeletal research, specifically around gluteal tendinopathy.

I confirm that I have read this submission and believe that I have an appropriate level of expertise to confirm that it is of an acceptable scientific standard, however I have significant reservations, as outlined above.

## References

[1] : Correlation of MRI findings with clinical findings of trochanteric pain syndrome. *Skeletal Radiol* .2008;37(10) : 10.1007/s00256-008-0514-8 903-9 10.1007/s00256-008-0514-8 18566811

[2] : Sonographic evaluation of gluteus medius and minimus tendinopathy. *Eur Radiol* .2003;13(6) : 10.1007/s00330-002-1740-4 1339-47 10.1007/s00330-002-1740-4 12764651

[3] : Sonography of greater trochanteric pain syndrome and the rarity of primary bursitis. *AJR Am J Roentgenol* .2013;201(5) : 10.2214/AJR.12.10038 1083-6 10.2214/AJR.12.10038 24147479

[4] : Prospective evaluation of magnetic resonance imaging and physical examination findings in patients with greater trochanteric pain syndrome. *Arthritis & Rheumatism* .2001;44(9) : 10.1002/1529-0131(200109)44:9<2138::AID-ART367>3.0.CO;2-M 2138-2145 10.1002/1529-0131(200109)44:9<2138::AID-ART367>3.0.CO;2-M 11592379

[5] : Increased substance P expression in the trochanteric bursa of patients with greater trochanteric pain syndrome. *Rheumatol Int* .2014;34(10) : 10.1007/s00296-014-2957-7 1441-8 10.1007/s00296-014-2957-7 24563019 PMC4856521

[6] : Does This Patient Have Hip Osteoarthritis?: The Rational Clinical Examination Systematic Review. *JAMA* .2019;322(23) : 10.1001/jama.2019.19413 2323-2333 10.1001/jama.2019.19413 31846019 PMC7583647

# Response

**Published:** 2024-06-09

**Authors:** Sania Almousa

**Affiliations:** Physiotherapy, Royal College of Surgeons in Ireland, Dublin, Ireland

Thank you very much for reviewing this protocol and for your insightful feedback. The protocol has been amended to address your comments. Please see below a point by point response to your comments.

 1. Thank you for your comments here. Apologies, there appears to have been some formatting errors with the citations, so some citations were incorrectly associated with the wrong text. Cook and Purdam (2009) citation has been removed.

 The following new citations have been added

- Fearon, A. M., Cook, J. L., Scarvell, J. M., Neeman, T., Cormick, W., & Smith, P. N. (2014). Greater trochanteric pain syndrome negatively affects work, physical activity and quality of life: a case control study. The Journal of arthroplasty, 29(2), 383-386.
- Connell, D.A., Bass, C., Sykes, C.J., Young, D. and Edwards, E., 2003. Sonographic evaluation of gluteus medius and minimus tendinopathy. *European radiology*, *13*(6), pp.1339-1347.
- Bird, P. A., Oakley, S. P., Shnier, R., & Kirkham, B. W. (2001). Prospective evaluation of magnetic resonance imaging and physical examination findings in patients with greater trochanteric pain syndrome. *Arthritis Rheum, 44*(9), 2138-2145.
- Long, S. S., Surrey, D. E., & Nazarian, L. N. (2013). Sonography of greater trochanteric pain syndrome and the rarity of primary bursitis. *Am J Roentgenol, 201*(5), 1083-1086.

2. A  new citation has been added

 Long, S.S., Surrey, D.E. and Nazarian, L.N., 2013. Sonography of greater trochanteric pain syndrome and the rarity of primary bursitis. *American Journal of Roentgenology*, *201*(5), pp.1083-1086.

 3. We have also replaced with this citation below

 Long, S.S., Surrey, D.E. and Nazarian, L.N., 2013. Sonography of greater trochanteric pain syndrome and the rarity of primary bursitis. *American Journal of Roentgenology*, *201*(5), pp.1083-1086.

 4. A new citation has been added

 Bird, P.A., Oakley, S.P., Shnier, R. and Kirkham, B.W., 2001. Prospective evaluation of magnetic resonance imaging and physical examination findings in patients with greater trochanteric pain syndrome. *Arthritis & Rheumatism: Official Journal of the American College of Rheumatology*, *44*(9), pp.2138-2145.

 5. Many thanks for this feedback. There appears to have been some formatting errors with the citations in version 1. We have reviewed all references to ensure that appropriate ones are used to support the text.

 6. A change has been done in the second paragraph

 Albers, I. S., Zwerver, J., Diercks, R. L., Dekker, J. H., & Van den Akker-Scheek, I. (2016). Incidence and prevalence of lower extremity tendinopathy in a Dutch general practice population: a cross sectional study. *BMC Musculoskelet Disord, 17*, 16.

 7. A change has been made.

 8. This has been reworded as follows

*‘’Although this evidence highlights the rationale for strengthening as a key intervention, traditionally, corticosteroid injections have been used to achieve pain relief. The lack of evidence for the long-term benefits of steroid injection* *(Mellor et al., 2018; Nissen et al., 2019; Rompe et al., 2009)* *highlights that interventions that result in long-term benefits are required. Additionally, steroid injections can  make tendons more vulnerable to tears* *(Abate, Salini, Schiavone, & Andia, 2017)* *.’’*

 9. The Rompe paper was chosen as it demonstrated that corticosteroid was less effective long-term than other conservative therapies. We have moved the citation in the sentence so that it is after part of the sentence which refers to the lack of evidence for long-term effects of steroid injection and the Nissen citation has also been added.

 10. Park, Ford and Kleinman references have been removed.

 The following reference has been added:

 Abate, M., Salini, V., Schiavone, C., & Andia, I. (2017). Clinical benefits and drawbacks of local corticosteroids injections in tendinopathies. *Expert Opin Drug Saf, 16*(3), 341-349. doi:10.1080/14740338.2017.1276561

 11. A change has been made in this paragraph in the Background section:

*‘’However, the 14 physiotherapy treatment sessions provided in the LEAP trial* *(Mellor et al., 2018) may not be feasible in physiotherapy practice.’’*

*12. *We have revised the wording to read as follows:

 ‘’However, the 14 physiotherapy treatment sessions provided in the LEAP trial (Mellor et al., 2018) may not be feasible in physiotherapy practice. For example, although exercise and education are the most common interventions for GT delivered by physiotherapists in Ireland, the number of physiotherapy sessions is usually limited to five or six (French, Grimaldi, Woodley, O'Connor, & Fearon, 2019). This treatment dosage is consistent across various musculoskeletal pathologies in the UK (Bury & Littlewood, 2018). Thus, whilst the LEAP trial in Australia demonstrated positive effects for EDX, delivery of this intervention over 14 sessions may not be practical to implement  into physiotherapy practice, and evaluation of a lower dose of face-to-face sessions with a healthcare practitioner, which could be implemented into clinical practice is warranted.’’

 13. Citations have been added:

 Mellor, R., Bennell, K., Grimaldi, A., Nicolson, P., Kasza, J., Hodges, P., . . . Vicenzino, B. (2018). Education plus exercise versus corticosteroid injection use versus a wait and see approach on global outcome and pain from gluteal tendinopathy: prospective, single blinded, randomised clinical trial. *BMJ, 361*, k1662.

 Wilson, R., Abbott, J. H., Mellor, R., Grimaldi, A., Bennell, K., & Vicenzino, B. (2023). Education plus exercise for persistent gluteal tendinopathy improves quality of life and is cost-effective compared with corticosteroid injection and wait and see: economic evaluation of a randomised trial. *J Physiother, 69*(1), 35-41.

 14. A change has been made:

 The wording ‘is essential to optimise exercise benefits’ has been removed so that the sentence reads as follows

 ‘ *’Completion of a daily Home Exercise Programme (HEP) is an integral part of the EDX-Ireland intervention.’’*

*15. *We have added the word ‘future’ into the aim to read as

 ‘ *We therefore aim to evaluate the feasibility of conducting a* *future* *definitive RCT, using a reduced dose (6 sessions) of a proven-effective physiotherapy treatment (14 sessions) consisting of EDucation plus eXercise for GT in an Irish context’*.

 We have also clarified the wording in relation to feasibility of conducting a future definitive RCT in the abstract.

 16. The primary outcome will be determined based on the data obtained from this trial. We have replaced the word ‘likely’ with ‘appropriate’ to read as follows:

 ‘’To estimate the effect size for an appropriate primary study outcome in a future definitive RCT’’ (Page 6)

 17. The symbol ‘’ +/- physical screening ‘’ has been removed. All participants who passed phone screening underwent physical screening procedure (Figure 1).

 18. The word ‘lateral’ has been added in to Table 1 - Inclusion criteria

 Reproduction of *lateral* hip pain on at least one of following

 diagnostic clinical tests:

 For diagnosis of hip OA, the following reference has been added in the reference list:

 Altman, R., Alarcon, G., Appelrouth, D., Bloch, D., Borenstein, D., Brandt, K., ... & Wolfe, F. (1991). The American College of Rheumatology criteria for the classification and reporting of osteoarthritis of the hip. *Arthritis & Rheumatism: Official Journal of the American College of Rheumatology*, *34*(5), 505-514.

 19. The citation by Blakenbaker et al (2008) has been added to Table 2.

*In addition, the following text has been added*

*‘’Criteria for tendon pathology on MRI as used by Mellor et al (2016) were adapted from Blankenbaker et al (2008) (Table 2 ).’’*

*20.* The paragraph has been reworded as follows:

*‘’Eligibility will be assessed through a 3-phase process of phone screening, physical screening and MRI.’’*

 AND

*‘’If participants meet the physical screening eligibility criteria, an MRI will be arranged to confirm the tendinopathic changes on MRI.’’*

*21. *The following text has been added:

*‘’A maximum of 5/10 on a pain NRS will be allowed as long as it does not result in increased pain that night or the next day. Other everyday activity loading that participants are undertaking, along with the exercise programme and technique, will be reviewed and modified accordingly to reduce loading levels.''*

 22. We have added the following sentence in the Usual Care paragraph:

*‘’The information leaflet is identical* *to* *that used in the LEAP Trial, however, no one-off education physiotherapy session will be provided for the Usual Care group.’’*

*The leaflet is included as been added to the extended data.*

*23. *We have added that 9 participants will be recruited from both EDX and usual care groups.

 We have clarified the number of treating therapists (n=7) and added the approximate number of referrers into the trial. who will participate in the qualitative interviews (approx 8-10). This is somewhat unknown as the optimal recruitment source for recruited patients is unknown and one of our feasibility objectives. (Page 13)

 24. The following as has been added to clarify that we used the same version as was used in the LEAP trial.

 ‘’Healthcare use and costs will be measured using a modified version of the *Osteoarthritis Cost and Consequences Questionnaire (OCC-Q)* (Pinto, Robertson, Hansen, & Abbott, 2011) as was used in LEAP Trial (Wilson et al., 2023); Mellor et al., 2018). Modifications included replacement of the wording ‘osteoarthritis’ with ‘hip pain’. Questions related to impact on work and previous joint replacement were removed.’’ (Page 16)

 25. The following sentence has been added in the adverse events paragraph:

*’’All adverse events will also be reported to the Trial Management Group (TMG) and the Trial Steering Committee (TSC).’’*

*26. *A new citation had been added in relation to the following statement:

 ‘ *’Participants will be identified by a study-specific unique identifier number (UIN) during the study. Processing of any personal data will comply with national and EU General Data Protection Regulations (GDPR), and compliance with GDPR will be maintained throughout the trial *(European Commission, 2018).’ *’*

*27. *Thank you for that comment. Recruitment rate refers to how many study participants are recruited per month to reach target sample size. This will inform planning of a future fully-powered trial in relation to the number of months required to recruit to target sample size.

 28. The following citation has been added:

 Albers, I. S., Zwerver, J., Diercks, R. L., Dekker, J. H., & Van den Akker-Scheek, I. (2016). Incidence and prevalence of lower extremity tendinopathy in a Dutch general practice population: a cross sectional study. *BMC Musculoskelet Disord, 17.*

*29. *The following change has been made:

*‘’Additionally, this study will quantify current usual care for this condition in Ireland.’’*

*30. *The written information leaflet provided to the ‘Usual Care’ group has been added to the Extended data with a DOI link.

 The education component in the 2018 LEAP trial entailed specific and detailed advice and education on tendon care. This information was delivered in hard copy handouts, verbal explanation and a DVD, which included an 18-minute education video.

 In the LEAP-Ireland trial, the exact same 18-minute video is being used and available for study participants in the PhysiApp software application, rather than by DVD, as well as verbal explanation. This education video is narrated by one of the LEAP investigators (Dr Grimaldi).

 Please see previous response in relation to the education component of the EDX intervention.

# Reviewer report: Reviewer response for version 1

**DOI:** 10.21956/hrbopenres.15095.r37534

**Published:** 2024-03-23

**Authors:** Chris Clifford, Katie Monnington

**Affiliations:** 1 Glasgow Caledonian University, Glasgow, UK; 2 Royal National Orthopaedic Hospital NHS Trust, London, UK

Reviewer 1:

**General Comments**

- Thank you for submitting your research protocol. This manuscript describes a feasibility randomised controlled trial (RCT) protocol where the authors intend to compare the effect of education plus exercise (EDX) to usual care in patients with gluteal tendinopathy. The study design is similar to a previous RCT published in the British Medical Journal [1]. Participants will be randomised to either i) 8 weeks of exercise and education or ii) ‘usual care’ which consists of a single session and exercise pamphlet. The aim of this study is to determine the feasibility of conducting an RCT before performing a larger definitive trial using a reduced dose (6 sessions of the EDX programme) that can potentially be implemented into existing health systems.

**Specific Comments**

- Is the ‘Usual Care’ group identical to the ‘Wait and See’ group in the LEAP trial? Could a sentence be added to clarify as this will likely be of interest to readers, especially those familiar with the LEAP trial?
- What will the ‘general advice’ given to the ‘Usual Care’ group consist of and what will be the duration of this session? For the LEAP trial the wait and see group received a 90-minute session [2]
- Is the written information leaflet provided to the ‘Usual Care’ group identical to what was previously provided to participant’s in the LEAP trial? Has it been amended based on authors experience/participant feedback/new developments in the field?
- Could the written information leaflet be added as an Appendix?
- Could the exercise programme be added as an Appendix with images?
- Do the authors plan to provide more detail on ‘pain on activity/loading’? While I understand it to be one of the ICON core domains, does this relate to walking, stairs, running?

Reviewer 2:

**General Comments**

 Thank you for the submission of your research protocol for a feasibility randomised controlled trial (RCT) exploring whether education plus exercise (EDX) compared to usual care in gluteal tendinopathy,  is effective at a lower dose of 6 sessions. The aim of this study will determine whether a further trial should be conducted to explore the effect of EDX at a lower dose to accommodate other health care systems. In addition a Study Within A Trial (SWAT) is planned to compare the recording of exercise adherence using app-based technology to a paper-based diary.

**Specific Comments:**

- Could there be further clarity on the rationale for the choice of 6 sessions in this study as difficulty gathering this information from the referenced article [3]
- Could the content of the training and education provided to the treating physiotherapists be included as an appendix?
- Terminology used for the exercises could be easily understood and in a common language. Are images of the exercises also planned to be included?
- Is the education component of EDX identical to that of LEAP 2018 [1]? Further clarity on the content and reasoning for the education component would therefore be helpful. Could this be added as an appendix?

Is the study design appropriate for the research question?

Yes

Is the rationale for, and objectives of, the study clearly described?

Yes

Are sufficient details of the methods provided to allow replication by others?

Yes

Are the datasets clearly presented in a useable and accessible format?

Not applicable

Reviewer Expertise:

Tendinopathy

We confirm that we have read this submission and believe that we have an appropriate level of expertise to confirm that it is of an acceptable scientific standard.

## References

[1] : Education plus exercise versus corticosteroid injection use versus a wait and see approach on global outcome and pain from gluteal tendinopathy: prospective, single blinded, randomised clinical trial. *BMJ* .2018;361: 10.1136/bmj.k1662 k1662 10.1136/bmj.k1662 29720374 PMC5930290

[2] : Perspectives and experiences of people who were randomly assigned to wait-and-see approach in a gluteal tendinopathy trial: a qualitative follow-up study. *BMJ Open* .2021;11(4) : 10.1136/bmjopen-2020-044934 e044934 10.1136/bmjopen-2020-044934 33883152 PMC8061810

[3] : Physiotherapy management of greater trochanteric pain syndrome (GTPS): an international survey of current physiotherapy practice. *Physiotherapy* .2020;109: 10.1016/j.physio.2019.05.002 111-120 10.1016/j.physio.2019.05.002 31493863

# Response

**Published:** 2024-06-09

**Authors:** Sania Almousa

**Affiliations:** Physiotherapy, Royal College of Surgeons in Ireland, Dublin, Ireland

Thank you very much for reviewing this protocol and for your insightful feedback. The protocol has been amended to address your comments. Please see below a point by point response to your comments.

 Reviewer 1:

 1. No, the ‘usual care’ group is not the same as the ‘wait and see group’ in the LEAP trial. The description of what usual care is outlined in the protocol in the usual care paragraph’ as follows as *’’treatment that a participant has followed so far or has been prescribed by their referrer into the trial (i.e., GP, orthopaedic consultant, rheumatologist or musculoskeletal triage physiotherapist). For those not referred from a healthcare practitioner (e.g., community recruitment/social media), usual care may not be relevant, but any healthcare utilisation will be recorded on a pre-standardised form.‘’*

*2. *No education session was provided to the ‘usual care’ group. Information was in the form of an information leaflet. The following has been added to the usual care section

*‘*’The information leaflet is identical to that used in the LEAP Trial, however, no one-off education physiotherapy session will be provided for the Usual Care group.’’

 3. The leaflet is identical to the one used in the LEAP Trial.

 We have added the following text in the Methods section (Usual Care paragraph)

*‘’The information leaflet is identical to that used in the LEAP Trial, however, no one-off education physiotherapy session will be provided for the Usual Care group.’’*

*4.*The written information leaflet provided to the ‘Usual Care’ group has been added to the Extended data with a DOI link.

 5. Images of the exercises with detailed descriptions are provided in the original LEAP trial publication by Mellor et al (2018) in a supplemental table. We have added the following text:

*‘’Additionally, the LEAP-Ireland trial will not entail use of a sliding platform with spring resistance during weeks 3-8; resistive exercise bands will be used to provide resistance. Details of the exercise programme with images, used in the LEAP trial are available in Supplemental Table 2 of the LEAP trial* *(Mellor et al., 2018)* *.’’*

 6. Pain on activity/loading relates to any activity/loading that is relevant to the participant, rather than pain at rest. As different activities could aggravate symptoms for different activities, pain with activity/loading was deemed more relevant for all study participants.

 We have added a sentence at the beginning of this section in the Methods detailing what the ‘wait and see’ comparator included in the LEAP trial as follows:

 ‘’ *In the LEAP trial '’wait and see’’ arm, participants attended one 30-minute session with a trial physiotherapist, where they received reassurance that the condition is likely to resolve over time, as well as advice regarding general tendon care and self-management* *(Mellor et al., 2016)*. *’’*

*Reviewer 2*

 1. Apologies, the information is in the related paper based on further responses to the survey across three countries: Australia, Ireland and New Zealand.

 ‘’French HP, Grimaldi A, Woodley SJ, O'Connor L, Fearon A. An international survey of current physiotherapy practice in diagnosis and knowledge translation of greater trochanteric pain syndrome (GTPS). Musculoskelet Sci Pract. 2019 Oct;43:122-126. doi: 10.1016/j.msksp.2019.06.002. Epub 2019 Jun 25. PMID: 31285186. ‘’

*2. *Images of the exercises with detailed descriptions are provided in the original LEAP trial publication by Mellor et al (2018) in a supplemental table. We have added the following text

 ‘’Additionally, the LEAP-Ireland trial will not entail use of a sliding platform with spring resistance during weeks 3-8; resistive exercise bands will be used to provide resistance. Details of the exercise programme with images, used in the LEAP trial are available in Supplemental Table 2 of the LEAP trial (Mellor et al., 2018).’’

*https://www.bmj.com/content/361/bmj.k1662*

*3. *Images of the exercises with detailed descriptions are provided in the original LEAP trial publication by Mellor et al (2018) in a supplemental table. We have added the following text

*‘’Additionally, the LEAP-Ireland trial did not entail use of a sliding platform with spring resistance during weeks 3-8; resistive exercise band was used to provide resistance. Details of the exercise programme with images, used in the LEAP trial are available in Supplemental Table 2 of the LEAP trial (Mellor et al, 2018). ‘’*

*4. *The education component explains relevant basic anatomy, terminology related to bursitis, tendinitis and tendinopathy, risk factors, sitting, sleeping and standing postures and walking advice and load modification

 The education component in the 2018 LEAP trial entailed specific and detailed advice and education on tendon care. This information was delivered in hard copy handouts, verbal explanation and a DVD, which included an education 18-minute video.

 In the LEAP-Ireland trial, the exact same 18-minute video is being used and available for study participants in the PhysiApp software application, rather than by DVD, as well as verbal explanation. This education video is narrated by one of the LEAP investigators (Dr Grimaldi).

 The following text has been added into the section on EDX in the Methods

*‘’This will be delivered via an 18-minute video, which was used in the LEAP trial (provided via DVD), which will be provided to study participants via the online Physiapp patient-facing companion application, used alongside the Physitrack online exercise prescription platform (* *https://www.physitrack.com/* *). The education video explains relevant basic anatomy, terminology related to tendinopathy and bursitis, risk factors, and advice related to sitting, lying positions walking and load modification. Participants will be asked to watch the education video prior to their first appointment with the physiotherapist.  Education will also be reinforced by the physiotherapist over the 8-week intervention period.’’*

 It is not possible to add this an appendix.
